# Ni–Rh-based bimetallic conductive MOF as a high-performance electrocatalyst for the oxygen evolution reaction

**DOI:** 10.3389/fchem.2023.1242672

**Published:** 2023-09-29

**Authors:** Shu Zu, Huan Zhang, Tong Zhang, Mingdao Zhang, Li Song

**Affiliations:** Jiangsu Collaborative Innovation Center of Atmospheric Environment, Jiangsu Key Laboratory of Atmospheric Environment Monitoring and Pollution Control, School of Environmental Science and Engineering, Nanjing University of Information Science and Technology, Nanjing, Jiangsu, China

**Keywords:** conductive metal–organic framework, active site, *in situ* growth, synergistic effect, oxygen evolution reaction

## Abstract

Metal–organic frameworks (MOFs) have recently been considered the promising catalysts due to their merits of abundant metal sites, versatile coordination groups, and tunable porous structure. However, low electronic conductivity of most MOFs obstructs their direct application in electrocatalysis. In this work, we fabricate an Ni–Rh bimetallic conductive MOF ([Ni_2.85_Rh_0.15_(HHTP)_2_]_*n*_/CC) grown *in situ* on carbon cloth. Abundant nanopores in the conductive MOFs expose additional catalytic active sites, and the advantageous 2D π-conjugated structure helps accelerate charge transfer. Owing to the introduction of Rh, [Ni_2.85_Rh_0.15_(HHTP)_2_]_*n*_/CC exhibited substantially improved oxygen evolution reaction (OER) activity and exhibited only an overpotential of 320 mV to achieve the current density of 20 mA cm^-2^. The remarkable OER performance confirmed by the electrochemical tests could be ascribed to the synergistic effect caused by the doped Rh together with Ni in [Ni_2.85_Rh_0.15_(HHTP)_2_]_*n*_/CC, thereby exhibiting outstanding electrocatalytic performance.

## 1 Introduction

With increasing energy consumption and global environmental concerns, renewable and clean energy technologies have been developed to support the sustainable development of human society (Cook et al., 2010; Chu and Majumdar, 2012; Roger et al., 2017; Seh et al., 2017). Electrocatalysis of the oxygen evolution reaction (OER) has been regarded as one of the important reactions for the conversion and storage technologies (Fu et al., 2017; Grimaud et al., 2017; Tahir et al., 2017; You and Sun, 2018). However, OER is a multi-step and four-electron reaction: 4OH^−^ (aq) → O_2_ (g) + 2H_2_O (l) + 4e^–^. Therefore, the kinetics are extremely sluggish, and large overpotentials are required to activate the reaction. In this case, high-activity electrocatalysts are required to overcome reaction barriers (Hunter et al., 2016; Grimaud et al., 2017; Suen et al., 2017; Tahir et al., 2017). Until now, the precious metals are still considered the typical OER catalysts. However, their widespread use is limited due to their low stability, excessive scarcity, and expensive cost (Tao et al., 2016; Suen et al., 2017; Wang et al., 2017; Bezerra and Maia, 2020). Consequently, it is vital to increase the efficiency and economics of OER electrocatalysts, hence supporting the growth of green energy (Hu and Dai, 2016; Wang et al., 2017; Bezerra and Maia, 2020). Generally, high-performance electrocatalysts should possess high conductivity, efficient proton transportability, long-term stability, and a high concentration of active sites. Modest adsorption free energy of OER reactive intermediates on the surface is particularly critical for improving the intrinsic activity of electrocatalysts (Seh et al., 2017; Suen et al., 2017).

Among several types of recently emerging functional materials, metal–organic frameworks (MOFs), a special type of crystalline porous materials have attracted immense attention for catalysis, owing to their enormous specific surface area and tunable pore dimensions (Motoyama et al., 2011; García-García et al., 2014; Wang et al., 2015; Guo et al., 2017; Park et al., 2018; Szczęśniak et al., 2018; Ma et al., 2020). While the MOFs have shown significant promise for electrochemical applications (Jahan et al., 2012; Furukawa et al., 2013; Shen et al., 2016; Guan et al., 2017; Zhang et al., 2017), the majority of them exhibit low electrical conductivity, severely limiting their electrocatalytic behavior and practical application as electrocatalysts (Xia et al., 2016; Zhao et al., 2016). While subjecting the MOFs to heat-treatment activation, the carbonization process can achieve good electrical conductivity but simultaneously cause absolute collapse of the well-defined pore/channel structures, thus transforming the active metal sites into the agglomerate phases and losing the pristine superiority. Recently, conductive MOFs have been proposed to exhibit superiority in both rapid charge transport (such as ligand–ligand π–π stacking) and high charge density (large concentration of charge carriers given by the loosely connected high-energy electrons from the metal nodes) (Kim et al., 2010; Zhang et al., 2013; Ding et al., 2015; Li C. et al., 2021). Due to the favorable properties of charge transfer, the conductive MOFs show the advantage of catalysis over the traditional MOFs for electrochemical reactions. However, the pristine single-metal conductive MOFs contain just a few redox-active centers, resulting in suboptimal electrocatalytic activity and stability. Therefore, the performance of pristine conductive MOFs is still far away from the commercial catalysts (Park et al., 2018). According to the previously reported works, bimetallic conductive MOFs displayed superior electrochemical property to the single-metal counterpart by tuning the conductivity, electronic structure, and absorption/desorption behaviors of intermediates (Zou et al., 2019; Chen et al., 2020; Li J. et al., 2021). Thus, incorporating different metal nodes into the frameworks to form bimetallic MOFs is a significant strategy for improving the catalytic activity of conductive MOFs (Giménez-Marqués et al., 2019). Recently, bimetallic systems have already demonstrated higher catalytic activity than monometallic counterparts, owing to their inherent properties (Yang et al., 2016; Yang and Xu, 2017; Yaqoob et al., 2021). It is reported that the conductive MOFs containing Co and Ni sites displayed superior ORR activity compared to their monometallic counterparts (only Co site or only Ni site) (Yoon et al., 2019). If loading a small amount of Fe into Ni-MOF-74, it required a lower overpotential and showed better OER activity, paving the way for the advancement of MOF-based electrocatalysts for direct employment (Gao et al., 2019). In this work, we make use of Rh to improve the electrochemical OER activity of Ni-based conductive MOFs.

2,3,6,7,10,11-Hexahydroxytriphenylene (HHTP) is a typical triphenylene-based organic ligand, with an abundance of functional -OH groups, which can be coordinated with metal atoms to form 2D planar π-conjugated MOFs. Due to the charge delocalization and orbital overlap between the metal node and organic linker, this kind of MOFs are characterized with intrinsic conductivity and can be directly used as the electrocatalyst without pyrolysis activation. Herein, we have designed and synthesized Ni–Rh-based bimetallic conductive MOFs [(Ni_x_Rh_y_)_3_(HHTP)_2_]_*n*_/CC using a simple one-step solvothermal method. Since the conductive MOFs were *in situ* securely grown on carbon cloth, the conductivity was greatly improved, and the electrochemical impedance was substantially reduced (Ali et al., 2018; Huang et al., 2020). It was discovered that the OER performance of [(Ni_x_Rh_y_)_3_(HHTP)_2_]_*n*_/CC was highly dependent on the Ni/Rh molar ratio. The optimized [Ni_2.85_Rh_0.15_(HHTP)_2_]_*n*_/CC showed the highest OER activity, with a low overpotential of 320 mV at the current density of 20 mA cm^-2^, similar to that of commercial RuO_2_/CC. The synergistic effect of Rh and Ni in [Ni_2.85_Rh_0.15_(HHTP)_2_]_*n*_/CC efficiently modulated the electronic state distribution of the two central metal atoms, hence altering the adsorption characteristics of oxygenous intermediates and improving the electrocatalytic activity and stability of OER.

## 2 Experimental

*Pretreatment of carbon cloth*: The carbon cloth (0.5 cm × 2 cm) was soaked in concentrated nitric acid for 24 h and then washed with deionized water until neutral. Subsequently, the carbon cloth samples were dried overnight in vacuum state at 60°C.

*Synthesis of [Ni*_*3*_ *HHTP*_*2*_ *]*_*n*_ */CC*: Cobalt acetate (Ni(OAc)_2_·4H_2_O, 13 mg) and hexahydroxytriphenylene (HHTP, 9.8 mg) were dissolved in 1.8 mL solution (deionized water: DMF = 5:1, v/v), followed by sonicating for 30 min. Afterward, the washed carbon cloth was put into the aforementioned solution. The reaction mixture was sealed into an autoclave, heated to 85°C, and maintained at this temperature for 12 h. After the autoclave was cooled down, the obtained carbon cloth was treated with deionized water and then freeze-dried for 24 h.

*Synthesis of [Ni_x_Rh_y_(HHTP)_2_]_n_/CC and [Ni_x_Rh_y_(HHTP)_2_]_n_ powder*: For the synthesis of [Ni_x_Rh_y_(HHTP)_2_]/CC, 0.7 mg/mL of rhodium acetate [Rh(OAc)_2_] solution was prepared ahead of time and mixed into Ni(OA_c_)_2_ and HHTP solution, according to the set ratio. Three different ratios of Ni/Rh were considered, the dosages of which are summarized in Supplementary Table S1. The synthetic steps of [Ni_x_Rh_y_(HHTP)_2_]_*n*_ powders were similar to those for [Ni_x_Rh_y_(HHTP)_2_]_*n*_/CC except without carbon cloth.

*Preparation of RuO*_*2*_ */CC*: The RuO_2_ dispersion solution was prepared by adding 20 μL of 5% Nafion solution and 4 mg RuO_2_ into 780 μL of mixed solution (H_2_O: ethanol = 1:1, v/v). Next, the RuO_2_ dispersion solution was drop-coated onto a 0.5 cm × 2 cm carbon cloth. After this step, the obtained carbon cloth with set RuO_2_ loading was dried overnight at 60°C.

*Measurements*: The phase composition of the sample was characterized by X-ray powder diffraction (XRD) using a Shimadzu XRD-6100 (Cu Kα radiation, *λ* = 1.5418 Å). The morphologies of samples were observed by transmission electron microscopy (TEM, JEM-2100F, Japan), and scanning electron microscopy (SEM) depictions were obtained using a Zeiss_Supra55 electron microscope. Fourier-transform infrared spectroscopy (FT-IR) was conducted in the range of 4,000–400 cm^-1^ on a Thermo Nicolet iS10 spectrometer. JEOL JEM 2100F was used for HRTEM and mapping. Raman spectra were recorded using HORIBA HR800 series.

*Electrochemical characterizations:* Electrochemical measurements were investigated using the electrochemical potentiostat (CHI 760E, Chenhua Instrument Company) in 0.1 M KOH at room temperature. The carbon cloth with the *in situ* grown catalyst was used as the working electrode directly. In addition, the platinum wire and the saturated calomel electrode were used as the counter and reference electrodes, respectively. All polarization curves were performed by linear voltammetric scan at a sweep rate of 5 mV s^-1^ and were iR-corrected. All potentials can be converted concerning the reversible hydrogen electrode (RHE), based on the following Nernst equation: E_(vs. RHE)_ = E_Ref_ + 0.059*13 + E_Test_. The electrochemical impedance spectroscopy (EIS) test was conducted on the working electrodes with an overpotential of 0.2 V, a frequency range of 0.01 ∼ 10^5^ Hz, and an amplitude of 5.0 mV.

## 3 Results and discussion

**SCHEME 1:**
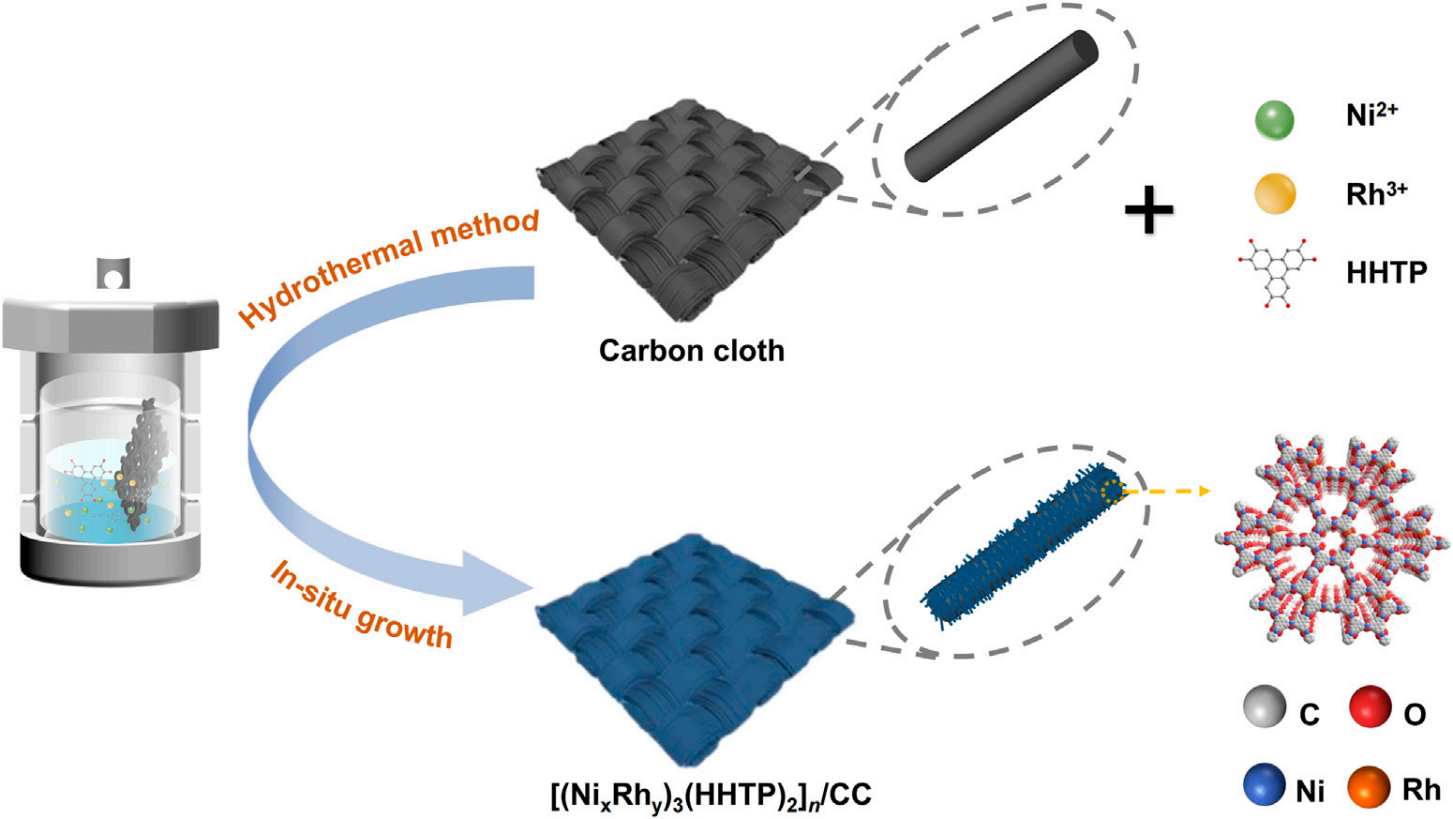
Schematic illustration of the *in situ* synthesis of [(Ni_x_Rh_y_(HHTP)_2_]_*n*_ on the carbon cloth.

**FIGURE 1:**
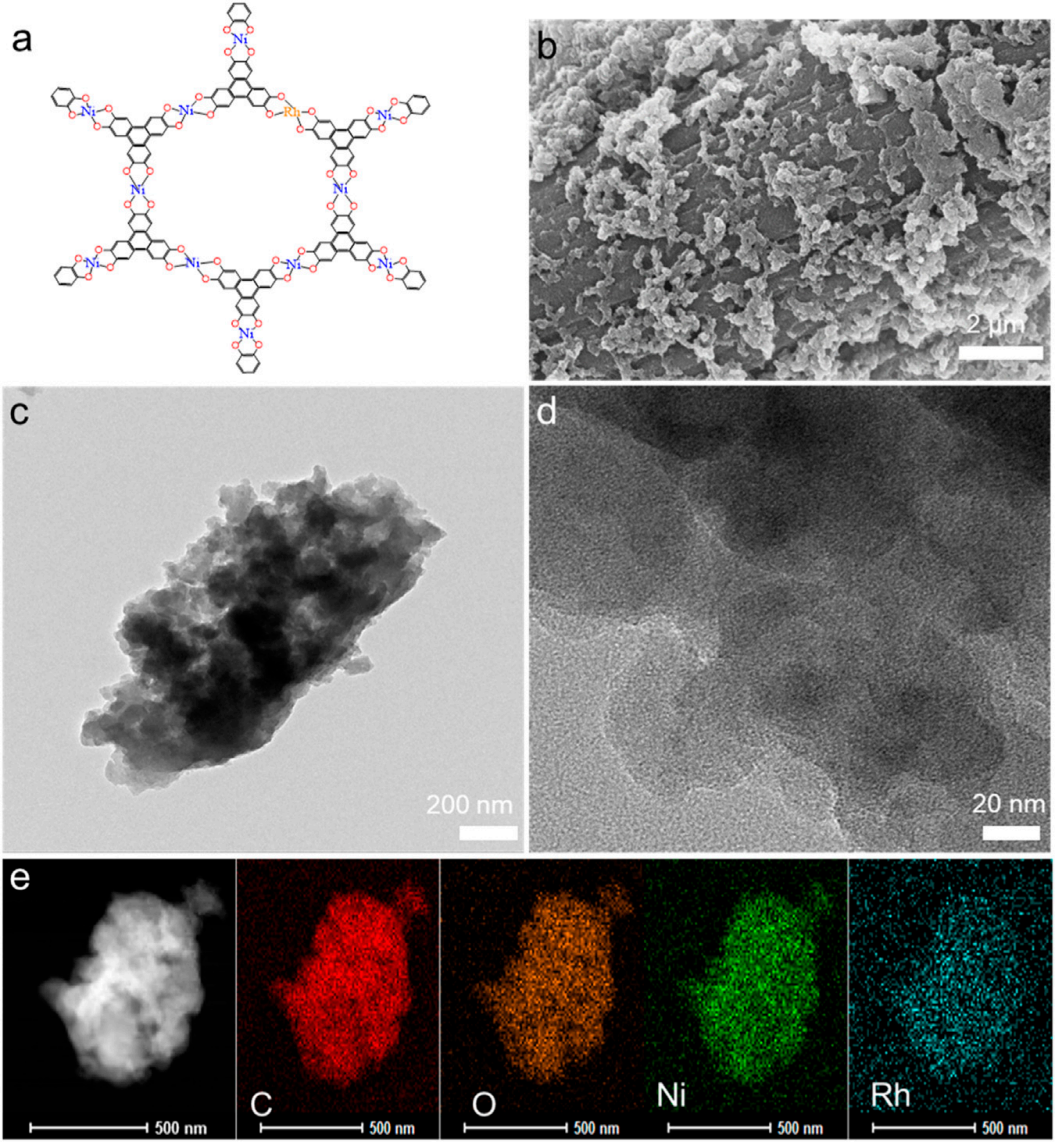
**(A)** Diagram of the coordination structure of Ni–Rh-based conductive MOF, **(B)** SEM image, **(C,D)** TEM images of [Ni_2.85_Rh_0.15_(HHTP)_2_]_*n*_/CC, and **(E)** dark-field image and the corresponding elemental mappings of C, O, Ni, and Rh in [Ni_2.85_Rh_0.15_(HHTP)_2_]_*n*_/CC.

The Ni–Rh-based bimetallic conductive MOF [(Ni_x_Rh_y_)_3_(HHTP)_2_]_*n*_ could be properly designed and synthesized with the one-step solvothermal method, which is schematically demonstrated in Scheme 1. During the solvothermal reaction process, the metallic ions could be coordinated by the ligands to fabricate a framework of long-range order. The chemical coordination and 3D structural modulation of Ni–Rh-based conductive MOF are displayed in Figure 1A and Supplementary Figure S1, respectively. The long-range-ordered planar structure enhances the structural integrity, and the typical p–p/p–d orbital overlaps impart the material with excellent electron transport capability. Because *in situ* growth process efficiently bonded these [Ni_x_Rh_y_(HHTP)_2_]_*n*_ MOFs to the backbone of the carbon cloth, they can be directly used as the OER electrodes. Differing from the smooth surface of the original carbon cloth (Supplementary Figures S2A, B), the surface of the manufactured material [Ni_3_(HHTP)_2_]_n_/CC displayed a homogeneous distribution of regular nanorods (Supplementary Figures S2C, D). However, when additional Rh was doped, the rod-like morphology of the resultant MOF crystals became irregular (Figure 1B and Supplementary Figures S2E–H). The transmission electron microscopy (TEM) images in Figure 1C showed that [Ni_2.85_Rh_0.15_(HHTP)_2_]_*n*_/CC had an irregular structure, suggesting that the doping of Rh had disrupted the process of crystallization and changed the rod-like morphology. As shown in Figure 1D, no hetero-structural phases existed, but a typical MOF morphology was observed in the view. Moreover, elemental mappings using the annular dark-field image corroborated the presence of C, O, Ni, and Rh, and these elements were distributed uniformly across the whole [Ni_2.85_Rh_0.15_(HHTP)_2_]_*n*_/CC surfaces (Figure 1E). These results indicated that the metal elements were successfully bonded with the ligands in the conductive MOFs.

**FIGURE 2:**
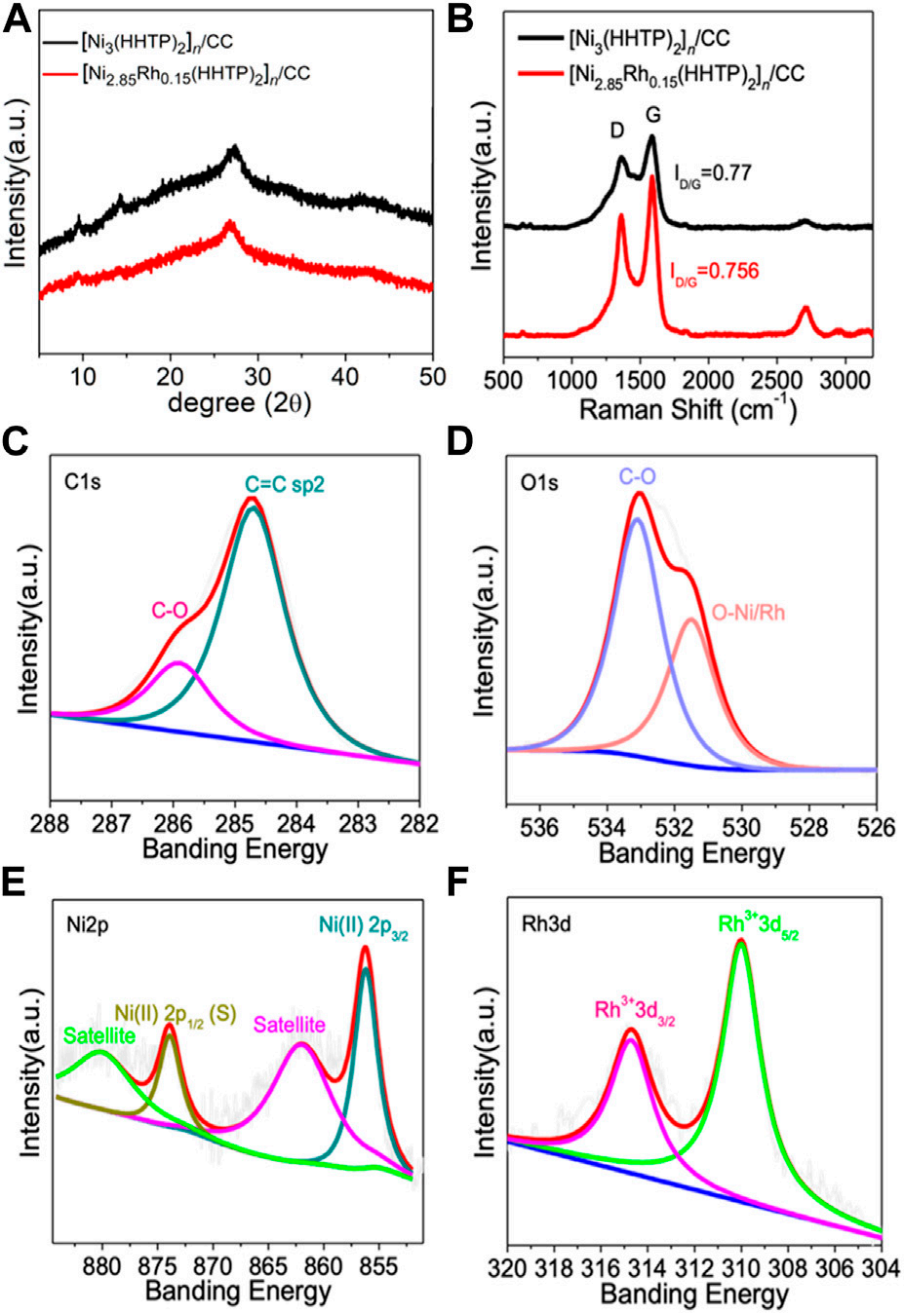
**(A)** XRD patterns of [Ni_3_(HHTP)_2_]_*n*_/CC and [Ni_2.85_Rh_0.15_(HHTP)_2_]_*n*_/CC, **(B)** Raman spectra of [Ni_3_(HHTP)_2_]_*n*_/CC and [Ni_2.85_Rh_0.15_(HHTP)_2_]_*n*_/CC, and high-resolution XPS spectra of **(C)** C 1s, **(D)** O 1s, **(E)** Ni 2p, and **(F)** Rh 3d in [[Ni_2.85_Rh_0.15_(HHTP)_2_]_*n*_/CC.

XRD was used to determine the phase and structure of [Ni_2.85_Rh_0.15_(HHTP)_2_]_*n*_/CC and [Ni_3_(HHTP)_2_]_*n*_/CC samples, as shown in Figure 2A. The results revealed that Rh doping did not appreciably alter the phase compositions and maintain the same crystal structure. In contrast, when Rh concentration increased gradually, the corresponding intensities of the XRD characteristic peak decreased significantly, implying that Rh replaced part of Ni to coordinate with HHTP, disturbing to a certain extent the structure of the MOFs (Supplementary Figure S3). After Rh doping, the functional groups contained in the samples remained intact, according to FT-IR (Supplementary Figure S4). The Raman spectra (Figure 2B) exhibited two wide peaks at ∼1,350 and ∼1,680 cm^-1^, which corresponded to the D and G peaks of the carbon material, respectively. The I_D_/I_G_ ratios obtained for [Ni_2.85_Rh_0.15_(HHTP)_2_]_*n*_/CC and [Ni_3_(HHTP)_2_]_*n*_/CC were 0.756 and 0.77, respectively, indicating that both [Ni_2.85_Rh_0.15_(HHTP)_2_]_*n*_/CC and [Ni_3_(HHTP)_2_]_*n*_/CC displayed the similar graphitized structure. This is because the graphitized structure is mainly ascribed to the carbon cloth, and the solvothermal reaction did not influence the property of the carbon cloth.

X-ray photoelectron spectroscopy (XPS) was adopted to investigate the surface chemistry and valence state within the prepared samples. The survey spectra of [Ni_2.85_Rh_0.15_(HHTP)_2_]_*n*_/CC (Supplementary Figure S5) displayed several obvious peaks assigned to C, O, Ni, and Rh. The high-resolution C 1s spectrum of [Ni_2.85_Rh_0.15_(HHTP)_2_]_*n*_/CC can be fitted into two characteristic peaks located at 284.6 eV and 286 eV, corresponding to C-O and C=C species, respectively (Figure 2C). Additionally, O-Ni/Rh (531.6 eV) and C–O (533 eV) were observed in the O 1s spectrum (Figure 2D), indicating that the ligand was successfully coordinated with Ni/Rh. These two characteristic peaks indicated the existence of the HHTP ligand, which was bound to the metal sites to form an ordered frame structure. As shown in Supplementary Figure S1, the coordination effect in the plane led to the generation of π-conjugate structures, which can reinforce the electron-transferring capability and therefore improve the catalytic performance (Zhang et al., 2021). The high-resolution Ni 2p spectrum (Figure 2E) demonstrated the typical characteristic peaks at the binding energies of 874 and 856 eV, which corresponded to Ni(II) 2p_1/2_ and Ni(II) 2p_3/2_, respectively, indicating the existence of Ni with a single valence state (Xu et al., 2021). The broader peaks located at 880 and 862 eV were assigned to the satellite peaks. In the high-resolution Rh 3d spectrum (Figure 2F), the two peaks at 314.7 and 310 eV correspond to Rh^3+^ 3d_3/2_ and Rh^3+^ 3d_5/2_, respectively, indicating that Rh existed in an trivalent form (Guo et al., 2019). In combination with the results of XRD, it was proved that the [Ni_2.85_Rh_0.15_(HHTP)_2_]_*n*_/CC sample has pure phase composition, without the formation of other heterogeneous phases (Pan et al., 2020; Qi et al., 2020; Pan et al., 2021).

**FIGURE 3:**
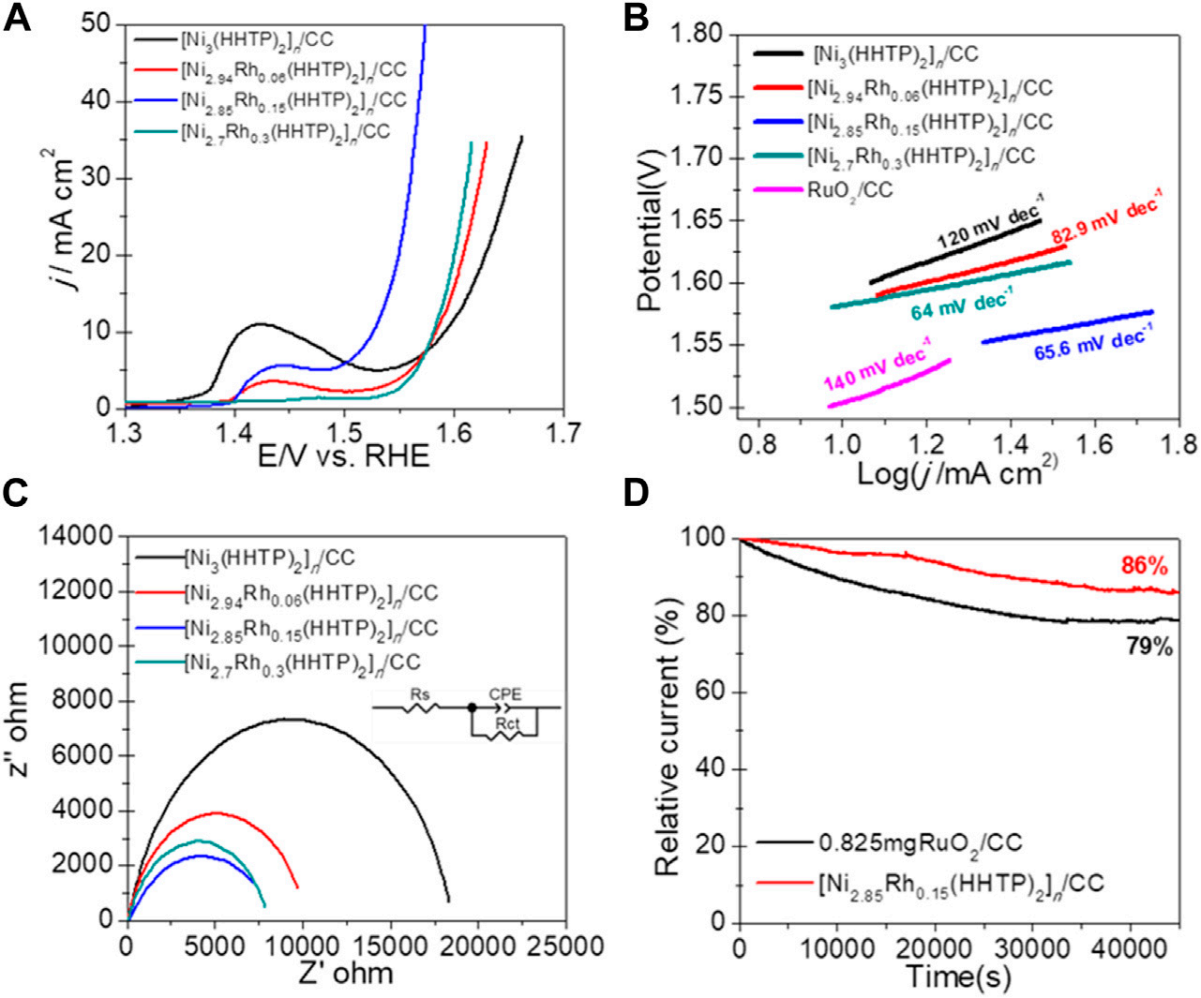
**(A)** LSV polarization curves and **(B)** related Tafel plots of [Ni_3_(HHTP)_2_]_*n*_/CC, [Ni_2.94_Rh_0.06_(HHTP)_2_]_*n*_/CC, [Ni_2.85_Rh_0.15_(HHTP)_2_]_*n*_/CC, [Ni_2.7_Rh_0.3_(HHTP)_2_]_*n*_/CC, and RuO_2_/CC for OER in 0.1 M KOH. **(C)** Nyquist plots of [Ni_3_(HHTP)_2_]_*n*_/CC, [Ni_2.94_Fe_0.06_(HHTP)_2_]_*n*_/CC, [Ni_2.85_Rh_0.15_(HHTP)_2_]_*n*_/CC, and [Ni_2.7_Rh_0.3_(HHTP)_2_]_*n*_/CC. **(D)** I–t chronoamperometric response of [Ni_2.85_Rh_0.15_(HHTP)_2_]_*n*_/CC and RuO_2_/CC. The loadings of [Ni_2.85_Rh_0.15_(HHTP)_2_]_*n*_/CC RuO_2_/CC were 0.825 mg.

The electrocatalytic activity of as-prepared conductive MOF electrocatalysts for OER was investigated in 0.1 M KOH solution using a typical three-electrode system. The linear sweep voltammetry (LSV) curves of the MOF samples with various Rh doping concentrations are shown in Figure 3A. We can find that the moderate doping of Rh might alter the electrochemical properties of [Ni_3_(HHTP)_2_]_*n*_/CC and therefore accelerate the OER process. However, excessive doping of Rh resulted in decreased OER activity, which was ascribed to the structure collapse and consequent conductivity attenuation of the conductive MOFs. [Ni_2.85_Rh_0.15_(HHTP)_2_]_*n*_/CC displayed the lowest potential of 1.55 V at 20 mA cm^−2^ compared to its single-metal counterpart (1.63 V for [Ni_3_(HHTP)_2_]_*n*_/CC) or other bimetallic conductive MOFs with different metal ratios (1.61 V for [Ni_2.94_Rh_0.06_(HHTP)_2_]_*n*_/CC and 1.6 V for [Ni_2.7_Rh_0.3_(HHTP)_2_]_*n*_/CC). It is noteworthy that the additional peak of 1.4 V, as shown in Figure 3A, was caused by the oxidation of Ni^2+^. It was agreeable to see in Supplementary Figure S6 that the bimetallic conductive MOFs of [Ni_2.85_Rh_0.15_(HHTP)_2_]_*n*_/CC displayed an excellent OER activity, even similar to that of RuO_2_/CC in terms of the potential at 20 mA cm^−2^, under the same mass loading of 0.825 mg. In addition, there was very poor electrocatalytic activity on the blank carbon cloth, implying that the excellent electrocatalytic performance was derived only from conductive bimetallic MOFs [Ni_2.85_Rh_0.15_(HHTP)_2_]_*n*_/CC (Cao et al., 2019). To further investigate the reaction kinetics, the corresponding Tafel slopes were analyzed. As shown in Figure 3B, the Tafel slopes of [Ni_3_(HHTP)_2_]_*n*_/CC, [Ni_2.94_Rh_0.06_(HHTP)_2_]_*n*_/CC, [Ni_2.85_Rh_0.15_(HHTP)_2_]_*n*_/CC, [Ni_2.7_Rh_0.3_(HHTP)_2_]_*n*_/CC, and RuO_2_/CC were 120, 82.9, 65.6, 64, and 140 mV dec^−1^, respectively. This result indicated that the bimetallic MOFs achieved faster reaction kinetics than the commercial RuO_2_ catalysts. In order to explore the charge transfer capability at the interface of the catalyst/electrolyte, the EIS test was performed. The Nyquist plots for different catalysts are provided in Figure 3C, in which the inset shows an equivalent circuit model. As demonstrated in Figure 3C, the Rh-doped MOFs exhibited much smaller charge transfer resistance (Rct) than the undoped [Ni_2.85_Rh_0.15_(HHTP)_2_]_*n*_/CC, indicating the doped Rh in favor of charge transfer during the OER process. In addition to the activity, long-term stability was another key concern for the practical application of electrocatalysts. Therefore, a chronoamperometry (I-t plot) response was performed at the constant potential of 1.6 V in 0.1 M KOH solution to assess the OER stability. Figure 3D shows that the [Ni_2.85_Rh_0.15_(HHTP)_2_]_*n*_/CC catalyst retained 86% of the initial current density after 45,000 s, which is larger than that of 79% for RuO_2_/CC, manifesting a better stability of [Ni_2.85_Rh_0.15_(HHTP)_2_]_*n*_/CC.

## 4 Conclusion

In summary, the conductive MOFs [Ni_2.85_Rh_0.15_(HHTP)_2_]_*n*_/CC were successfully synthesized with a simple solvothermal method. The obtained conductive MOF showed prominent OER activity comparable to RuO_2_/CC and outperformed RuO_2_/CC in terms of long-term stability. The remarkable activity could be attributed to the abundant active metallic sites in the MOFs, which are in favor of reactant adsorption and activation. The porous morphology leads to high-efficient mass transfer and ion penetration. In addition, the conductive characteristics of the MOFs facilitate charge transfer during the OER. The multi-aspect merits synergistically improve the electrocatalytic activity. Therefore, this work provides a facile method to synthesize the bimetallic conductive MOFs, being directly used as the efficient OER electrocatalysts.

## Data Availability

The original contributions presented in the study are included in the article/Supplementary Material; further inquiries can be directed to the corresponding author.
