# Supplementary material for: Ni–Rh-based bimetallic conductive MOF as a high-performance electrocatalyst for the oxygen evolution reaction
Source: Front Chem. 2023 Sep 29;11:1242672. doi: 10.3389/fchem.2023.1242672 (PMC10570521; doi:10.3389/fchem.2023.1242672)
Supplement: Supplementary file 1 [file DataSheet1.docx]

**Ni-Rh-based Bimetallic Conductive MOF as a High-performance Electrocatalyst for Oxygen Evolution Reaction**

Shu Zu, Huan Zhang, Tong Zhang, Mingdao Zhang, Li Song*

*School of Environmental Science and Engineering, Jiangsu Key Laboratory of Atmospheric Environment Monitoring and Pollution Control, Jiangsu Collaborative Innovation Center of Atmospheric Environment, Nanjing University of Information Science & Technology, Nanjing, 210044, Jiangsu, P. R. China.*

E-mail: songli@nuist.edu.cn.


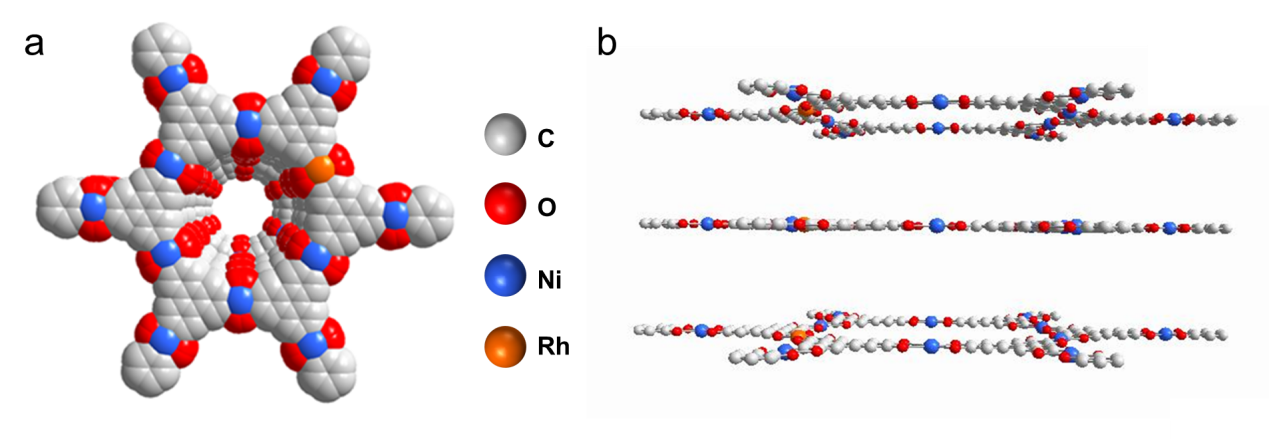


**Fig. S1**. (a) View of the [(NiRh)_3_(HHTP)_2_]*_n_* structure along the channels, (b) View of the two extended corrugated layers along the [110] direction.


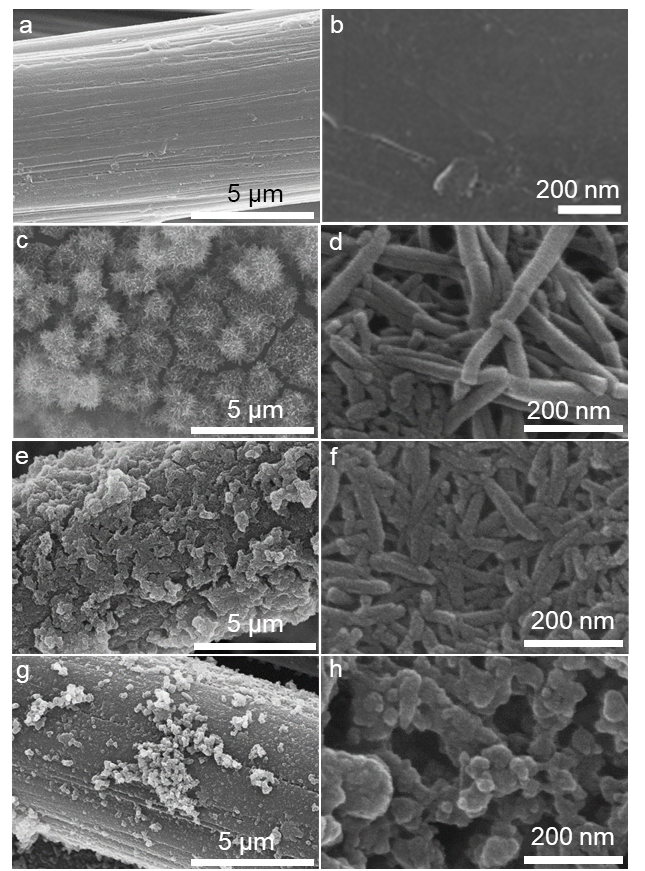


**Fig. S2**. SEM images of (a, b) bare carbon cloth, (c, d) [Ni_3_(HHTP)_2_]*_n_*/CC, (e, f) [Ni_2.94_Rh_0.06_(HHTP)_2_]*_n_*/CC, (g, h) [Ni_2.7_Rh_0.3_(HHTP)_2_]*_n_*/CC.


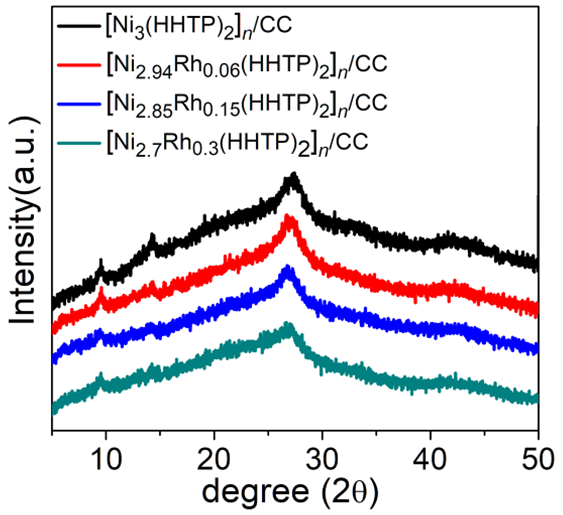


**Fig. S3.** XRD patterns of [Ni_3_(HHTP)_2_]*_n_*, [Ni_2.94_Fe_0.06_(HHTP)_2_]*_n_*/CC, [Ni_2.85_Rh_0.15_(HHTP)_2_]*_n_*/CC and [Ni_2.7_Rh_0.3_(HHTP)_2_]*_n_*/CC.


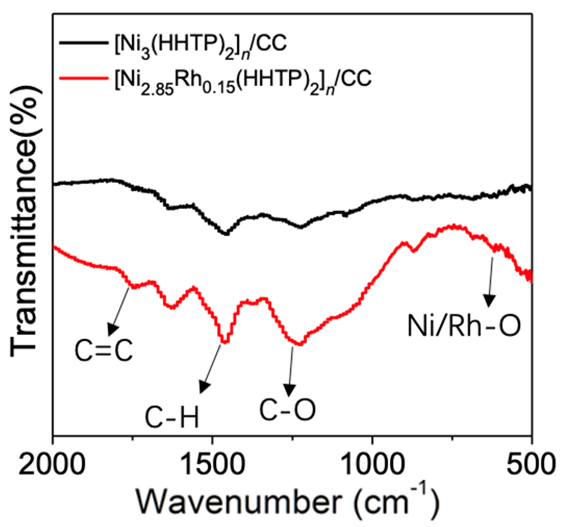


**Fig. S4.** FTIR spectra of [Ni_3_(HHTP)_2_]*_n_* /CC, [Ni_2.85_Rh_0.15_(HHTP)_2_]*_n_*/CC.


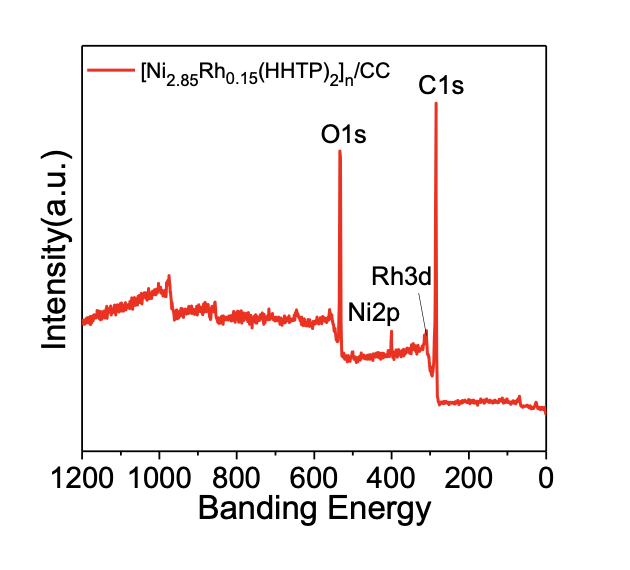


**Fig. S5.** XPS spectra of [Ni_2.85_Rh_0.15_(HHTP)_2_]*_n_*/CC.

**Fig. S6.** The LSV polarization curves of [Ni_2.85_Rh_0.15_(HHTP)_2_]*_n_*/CC, RuO_2_/CC and CC for OER in 0.1M KOH.

**Table S1.** The addition amount of raw reactants

|  | Ni(OAc)_2_/mg | Rh(OAc)_2_(0.74mg/ml) | HHTP/mg | H_2_O/ml | DMF/ml |
| --- | --- | --- | --- | --- | --- |
| [Ni_3_(HHTP)_2_]*_n_*/CC | 11.28 | / | 9.8 | 1.5 | 0.3 |
| [Ni_2.94_Rh_0.06_(HHTP)_2_]*_n_*/CC | 11.0544 | 0.2 | 9.8 | 1.3 | 0.3 |
| [Ni_2.85_Rh_0.15_(HHTP)_2_]*_n_*/CC | 10.716 | 0.5 | 9.8 | 1 | 0.3 |
| [Ni_2.7_Rh_0.3_(HHTP)_2_]*_n_*/CC | 10.152 | 1.0 | 9.8 | 0.5 | 0.3 |
